# *N*,*N*′-[(1*S*,2*S*)-Cyclo­hexane-1,2-di­yl]bis­(4-methyl­benzene­sulfonamide)

**DOI:** 10.1107/S1600536811012372

**Published:** 2011-04-13

**Authors:** Yi-Ling Hong, Hua-Jie Tan, Liang Shen

**Affiliations:** a College of Material Chemistry and Chemical Engineering, Hangzhou Normal University, Hangzhou, People’s Republic of China

## Abstract

In the title compound, C_20_H_26_N_2_O_4_S_2_, the cyclo­hexane ring has a chair conformation. The two chiral C atoms are in *S* configurations. In the crystal, inter­molecular N—H⋯O hydrogen bonds link the mol­ecules into chains propagating in [001]. Weak inter­molecular C—H⋯O hydrogen bonds further stabilize the crystal packing.

## Related literature

For the preparation of the title compound, see: Guo *et al.* (1997 ▶). For asymmetric catalysis, see: Ackermann *et al.* (2003 ▶); Bisai *et al.* (2005 ▶); Costa *et al.* (2005 ▶); Schwarz *et al.* (2010 ▶). For the crystal structures of racemates of the title compound, see: Nieger *et al.* (2004 ▶); Pritchett *et al.* (1999 ▶); Tasker *et al.* (2005 ▶). 
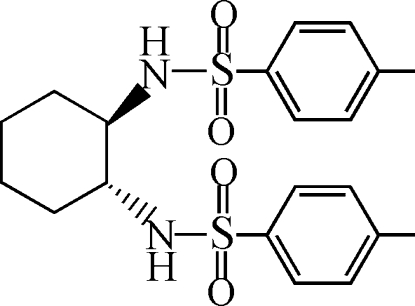


## Experimental

### 

#### Crystal data

C_20_H_26_N_2_O_4_S_2_
*M* *_r_* = 422.55
Orthorhombic, 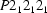

*a* = 11.5704 (14) Å
*b* = 12.2585 (15) Å
*c* = 15.3757 (19) Å
*V* = 2180.8 (5) Å^3^
*Z* = 4
Mo *K*α radiation
μ = 0.27 mm^−1^
*T* = 296 K
0.75 × 0.65 × 0.32 mm

#### Data collection

Bruker SMART CCD diffractometer
Absorption correction: multi-scan (*SADABS*; Sheldrick, 1996 ▶) *T*_min_ = 0.822, *T*_max_ = 0.918
9486 measured reflections
4196 independent reflections
3748 reflections with *I* > 2σ(*I*)
*R*_int_ = 0.021

#### Refinement

*R*[*F* ^2^ > 2σ(*F* ^2^)] = 0.035
*wR*(*F* ^2^) = 0.094
*S* = 1.00
4196 reflections
256 parameters
H-atom parameters constrained
Δρ_max_ = 0.18 e Å^−3^
Δρ_min_ = −0.24 e Å^−3^
Absolute structure: Flack (1983 ▶), 1706 Friedel pairs
Flack parameter: 0.07 (7)

### 

Data collection: *SMART* (Bruker, 1998 ▶); cell refinement: *SAINT* (Bruker, 1998 ▶); data reduction: *SAINT*; program(s) used to solve structure: *SHELXS97* (Sheldrick, 2008 ▶); program(s) used to refine structure: *SHELXL97* (Sheldrick, 2008 ▶); molecular graphics: *ORTEP-3* (Farrugia,1997 ▶); software used to prepare material for publication: *WinGX* (Farrugia, 1999 ▶).

## Supplementary Material

Crystal structure: contains datablocks I, global. DOI: 10.1107/S1600536811012372/cv5062sup1.cif

Structure factors: contains datablocks I. DOI: 10.1107/S1600536811012372/cv5062Isup2.hkl

Additional supplementary materials:  crystallographic information; 3D view; checkCIF report

## Figures and Tables

**Table 1:** Hydrogen-bond geometry (Å, °)

*D*—H⋯*A*	*D*—H	H⋯*A*	*D*⋯*A*	*D*—H⋯*A*
N1—H101⋯O3^i^	0.96	2.02	2.971 (3)	171
N2—H102⋯O1^ii^	0.93	2.07	2.982 (3)	167
C11—H11⋯O4^iii^	0.93	2.55	3.214 (3)	129
C9—H9⋯O1^iv^	0.93	2.54	3.452 (3)	169

Symmetry codes: (i) 
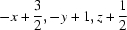
; (ii) 
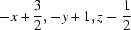
; (iii) 
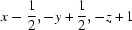
; (iv) 
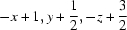
.

**Fig. 1.**
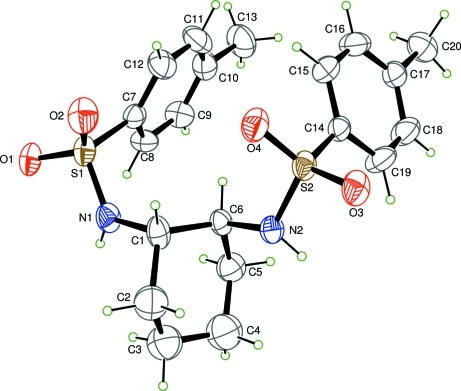
Molecular structure of (I) showing 40% probability displacement ellipsoids.

## supplementary crystallographic information

### Comment

Chiral bis(sulfonamide)-based ligands have been successfully used in a variety of catalytic asymmetric transformations, such as asymmetric Diels-Alder cycloaddition, [2 + 2]cycloaddition, Claisen rearrangement, enolization-amination reactions, the cyclopropanation of allylic alcohols and the addition of alkyl groups to aldehydes. Among the above-mentioned reactions, the asymmetric addition of alkyl groups to aldehydes is one of the most efficient and highly enantioselective carbon-carbon bond forming processes (Ackermann *et al.*, 2003; Costa *et al.*, 2005). Bis(sulfonamide)-based ligands exhibit efficiency and enantioselectivity in the field of asymmetric synthesis, due to the robust nature of this linkage and bind well to some metals (Bisai *et al.*, 2005; Schwarz *et al.*, 2010). However, little was known about the structure of chiral bis(sulfonamide) ligands and, therefore, about the structure-catalytic activity relationships. Herein, we report the synthesis and crystal structure of the title compound (I) - a chiral bis(sulfonamide)-based ligand.

In (I) (Fig. 1), the  C—C sigma single bond lengths in cyclohexane ring fall in the 1.478 (7) to 1.530 (3)Å range. The C1—C6 distance is 1.530 (3) Å, which is slightly longer than the corresponding distances of C1—C2 (1.508 (3) Å) and C5—C6 (1.524 (5) Å) resulting from the possible electron-withdrawing nature of the sulfonamide groups. The S1—O1 bond lengths of 1.4401 (16)Å is longer than other S1—O2 distances(1.4212 (17) Å), and S2—O3 distance (1.4376 Å) is also longer than S2—O4 bond lengths(1.4212 (19) Å). The disparity is a result of the forming of the hydrogen bonds involving O1 atom and O3. The bond lengths of S1—N1, S2—N2, S1—C7 and S2—C14 are 1.616 (19), 1.597 (2), 1.751 (3) and 1.769 (2) Å, which are comparable with these in racemic *N*,*N*'-cyclohexane-1,2-diylbis(4-methylbenzenesulfonamide) (Pritchett *et al.*, 1999; Nieger *et al.*, 2004; Tasker *et al.*, 2005). The bond angles involving the O atoms involved in hydrogen-bonding, N1—S1—O1 and N2—S2—O3 are 104.81 (10) and 105.48 (11)°, respectively, while N1—S1—O2 and N2—S2—O4 are 108.82 (11) and 108.47 (12)°, respectively. The C—C—C bond angles within the cyclohexane rings are in the range 109.9 (3)–112.3 (3)°.

In the crystal structure, intermolecular N—H···O hydrogen bonds (Table 1) link the molecules into chains propagated in [001]. Weak intermolecular C—H···O hydrogen bonds (Table 1) stabilize further the crystal packing.

### Experimental

*N*,*N*'-(1*S*,2S)-Cyclohexane-1,2-diylbis(4- methylbenzenesulfonamide) was prepared according to literature method (Guo *et al.*, 1997). To a stirred solution of (1*S*,2S)-1,2-diaminocyclohexane(1.2950 g, 11.36 mmol) in THF(100 mL) was added triethylamine(4.7 mL, 34 mmol) and the mixture was cooled to 0¯C and a solution of *p*-toluene sulfonyl chloride(4.3815 g, 22.72 mmol) in THF(10 mL) was added dropwise over 0.5–1 h. After the addition was complete, the mixture was allowed to warm to room temperature and stirred for 12 h. Then, the solvent removed under reduced pressure to obtain crude product. The crude product resolved in dichloromethane(10 mL), and washed with saturated sodium carbonate (13.5 mL). The aqueous solution was then extracted with dichloromethane(30 mL). The dichloromethane layers were combined, dried over anhydrous Na_2_SO_4_, filtered, and obtained title compound. The compound was characterized by elemental analysis, IR, 1*H*-NMR and MS. Yellow crystals suitable for X-ray diffraction were grown from hexane/ethyl acetate as a solvent.

### Refinement

The amino H atoms were located in a difference Fourier map and refined as riding, with *U*_iso_(H) = 1.2*U*_eq_(N). The remaining H atoms were placed in a calculated positions with C—H = 0.93–0.98Å and were included in the final cycle of refinement in riding mode with *U*_iso_(H) = 1.2 or 1.5*U*_eq_(C).

### Crystal data

**Table:**

C_20_H_26_N_2_O_4_S_2_	*F*(000) = 896
*M**_r_* = 422.55	*D*_x_ = 1.287 Mg m^−^^3^
Orthorhombic, *P*2_1_2_1_2_1_	Mo *K*α radiation, λ = 0.71073 Å
Hall symbol: P 2ac 2ab	Cell parameters from 5610 reflections
*a* = 11.5704 (14) Å	θ = 2.2–27.4°
*b* = 12.2585 (15) Å	µ = 0.27 mm^−^^1^
*c* = 15.3757 (19) Å	*T* = 296 K
*V* = 2180.8 (5) Å^3^	Chunk, yellow
*Z* = 4	0.75 × 0.65 × 0.32 mm

### Data collection

**Table:**

Bruker SMART CCD diffractometer	4196 independent reflections
Radiation source: fine-focus sealed tube	3748 reflections with *I* > 2σ(*I*)
graphite	*R*_int_ = 0.021
φ and ω scans	θ_max_ = 26.0°, θ_min_ = 2.1°
Absorption correction: multi-scan (*SADABS*; Sheldrick, 1996)	*h* = −14→14
*T*_min_ = 0.822, *T*_max_ = 0.918	*k* = −15→7
9486 measured reflections	*l* = −18→17

### Refinement

**Table:**

Refinement on *F*^2^	Hydrogen site location: inferred from neighbouring sites
Least-squares matrix: full	H-atom parameters constrained
*R*[*F*^2^ > 2σ(*F*^2^)] = 0.035	*w* = 1/[σ^2^(*F*_o_^2^) + (0.0445*P*)^2^ + 0.4906*P*] where *P* = (*F*_o_^2^ + 2*F*_c_^2^)/3
*wR*(*F*^2^) = 0.094	(Δ/σ)_max_ < 0.001
*S* = 1.00	Δρ_max_ = 0.18 e Å^−^^3^
4196 reflections	Δρ_min_ = −0.24 e Å^−^^3^
256 parameters	Extinction correction: *SHELXL97* (Sheldrick, 2008), Fc^*^=kFc[1+0.001xFc^2^λ^3^/sin(2θ)]^-1/4^
0 restraints	Extinction coefficient: 0.0129 (10)
Primary atom site location: structure-invariant direct methods	Absolute structure: Flack (1983), 1706 Friedel pairs
Secondary atom site location: difference Fourier map	Flack parameter: 0.07 (7)

### Special details

**Table:**

Geometry. All e.s.d.'s (except the e.s.d. in the dihedral angle between two l.s. planes) are estimated using the full covariance matrix. The cell e.s.d.'s are taken into account individually in the estimation of e.s.d.'s in distances, angles and torsion angles; correlations between e.s.d.'s in cell parameters are only used when they are defined by crystal symmetry. An approximate (isotropic) treatment of cell e.s.d.'s is used for estimating e.s.d.'s involving l.s. planes.
Refinement. Refinement of *F*^2^ against ALL reflections. The weighted *R*-factor *wR* and goodness of fit *S* are based on *F*^2^, conventional *R*-factors *R* are based on *F*, with *F* set to zero for negative *F*^2^. The threshold expression of *F*^2^ > σ(*F*^2^) is used only for calculating *R*-factors(gt) *etc*. and is not relevant to the choice of reflections for refinement. *R*-factors based on *F*^2^ are statistically about twice as large as those based on *F*, and *R*- factors based on ALL data will be even larger.

### Fractional atomic coordinates and isotropic or equivalent isotropic displacement parameters (Å^2^)

**Table:**

	*x*	*y*	*z*	*U*_iso_*/*U*_eq_	
S2	0.67936 (6)	0.34616 (4)	0.33373 (4)	0.05778 (17)	
S1	0.62809 (5)	0.40875 (4)	0.66645 (3)	0.05630 (16)	
C14	0.54709 (19)	0.41739 (18)	0.34420 (13)	0.0522 (5)	
O1	0.66322 (17)	0.40834 (14)	0.75630 (9)	0.0696 (5)	
O4	0.6663 (2)	0.24457 (14)	0.37717 (13)	0.0815 (6)	
C7	0.4973 (2)	0.48007 (17)	0.65910 (13)	0.0546 (5)	
O3	0.70891 (17)	0.34735 (14)	0.24292 (10)	0.0691 (5)	
N2	0.77908 (17)	0.41378 (18)	0.38123 (10)	0.0600 (5)	
H102	0.8017	0.4755	0.3500	0.090*	
N1	0.72602 (17)	0.47945 (16)	0.61697 (10)	0.0553 (5)	
H101	0.7469	0.5406	0.6526	0.083*	
O2	0.61429 (19)	0.30761 (13)	0.62249 (11)	0.0717 (5)	
C1	0.71703 (19)	0.49569 (17)	0.52189 (12)	0.0486 (5)	
H1	0.6373	0.4812	0.5038	0.058*	
C10	0.2880 (2)	0.5933 (2)	0.65482 (15)	0.0641 (6)	
C13	0.1751 (3)	0.6529 (3)	0.6547 (2)	0.0824 (8)	
H13A	0.1154	0.6058	0.6764	0.124*	
H13B	0.1565	0.6749	0.5964	0.124*	
H13C	0.1808	0.7163	0.6911	0.124*	
C19	0.5374 (2)	0.5203 (2)	0.30984 (16)	0.0624 (6)	
H19	0.6004	0.5527	0.2825	0.075*	
C11	0.2962 (2)	0.4872 (2)	0.62491 (19)	0.0752 (7)	
H11	0.2306	0.4529	0.6030	0.090*	
C8	0.4911 (2)	0.5865 (2)	0.68795 (18)	0.0765 (7)	
H8	0.5567	0.6212	0.7093	0.092*	
C2	0.7466 (3)	0.6124 (2)	0.50033 (16)	0.0815 (9)	
H2A	0.6980	0.6607	0.5345	0.098*	
H2B	0.7301	0.6258	0.4394	0.098*	
C6	0.7972 (2)	0.4147 (2)	0.47615 (13)	0.0631 (6)	
H6	0.7820	0.3414	0.4989	0.076*	
C18	0.4338 (2)	0.5754 (2)	0.31602 (18)	0.0728 (7)	
H18	0.4279	0.6455	0.2933	0.087*	
C12	0.3992 (3)	0.4310 (2)	0.62680 (17)	0.0719 (7)	
H12	0.4025	0.3597	0.6062	0.086*	
C17	0.3394 (2)	0.5287 (2)	0.35501 (17)	0.0723 (7)	
C15	0.4543 (3)	0.3685 (2)	0.38376 (17)	0.0733 (7)	
H15	0.4605	0.2985	0.4067	0.088*	
C20	0.2257 (3)	0.5907 (3)	0.3615 (2)	0.1059 (11)	
H20A	0.2108	0.6088	0.4212	0.159*	
H20B	0.1641	0.5460	0.3396	0.159*	
H20C	0.2305	0.6564	0.3277	0.159*	
C9	0.3878 (3)	0.6408 (2)	0.6850 (2)	0.0810 (8)	
H9	0.3850	0.7128	0.7041	0.097*	
C16	0.3510 (3)	0.4257 (3)	0.38880 (19)	0.0850 (8)	
H16	0.2878	0.3933	0.4159	0.102*	
C5	0.9229 (3)	0.4441 (5)	0.4944 (2)	0.1246 (19)	
H5A	0.9394	0.4311	0.5554	0.149*	
H5B	0.9727	0.3968	0.4604	0.149*	
C4	0.9504 (3)	0.5625 (6)	0.4727 (2)	0.156 (3)	
H4A	1.0295	0.5782	0.4892	0.187*	
H4B	0.9435	0.5733	0.4104	0.187*	
C3	0.8719 (4)	0.6387 (4)	0.5182 (2)	0.1297 (18)	
H3A	0.8881	0.7126	0.4992	0.156*	
H3B	0.8861	0.6348	0.5803	0.156*	

### Atomic displacement parameters (Å^2^)

**Table:**

	*U*^11^	*U*^22^	*U*^33^	*U*^12^	*U*^13^	*U*^23^
S2	0.0767 (4)	0.0523 (3)	0.0444 (3)	0.0109 (3)	0.0052 (3)	−0.0021 (2)
S1	0.0786 (4)	0.0508 (3)	0.0395 (2)	−0.0039 (3)	0.0075 (3)	0.0023 (2)
C14	0.0620 (13)	0.0541 (11)	0.0406 (10)	−0.0011 (10)	−0.0020 (9)	−0.0021 (10)
O1	0.0994 (13)	0.0680 (9)	0.0414 (8)	−0.0018 (10)	0.0051 (8)	0.0113 (7)
O4	0.1114 (16)	0.0534 (9)	0.0798 (12)	0.0169 (10)	0.0114 (11)	0.0073 (8)
C7	0.0707 (14)	0.0495 (10)	0.0437 (11)	−0.0136 (10)	0.0092 (11)	−0.0052 (9)
O3	0.0919 (13)	0.0705 (10)	0.0449 (8)	0.0055 (10)	0.0063 (8)	−0.0122 (7)
N2	0.0655 (12)	0.0763 (12)	0.0382 (9)	0.0112 (11)	0.0029 (8)	0.0005 (9)
N1	0.0646 (11)	0.0676 (11)	0.0337 (8)	−0.0030 (9)	0.0017 (8)	0.0045 (8)
O2	0.0989 (14)	0.0533 (8)	0.0631 (10)	0.0004 (9)	0.0087 (10)	−0.0038 (8)
C1	0.0517 (11)	0.0612 (11)	0.0330 (9)	0.0032 (9)	−0.0022 (8)	0.0039 (9)
C10	0.0732 (15)	0.0639 (13)	0.0551 (13)	−0.0078 (12)	0.0077 (11)	0.0031 (11)
C13	0.0744 (17)	0.0878 (18)	0.085 (2)	−0.0004 (15)	0.0022 (15)	0.0123 (16)
C19	0.0597 (13)	0.0589 (13)	0.0687 (15)	−0.0012 (11)	0.0004 (11)	0.0099 (11)
C11	0.0691 (17)	0.0690 (15)	0.0875 (18)	−0.0214 (14)	−0.0050 (15)	−0.0062 (14)
C8	0.0723 (17)	0.0602 (14)	0.097 (2)	−0.0060 (13)	−0.0110 (15)	−0.0275 (14)
C2	0.130 (3)	0.0718 (16)	0.0428 (12)	−0.0146 (16)	−0.0041 (14)	0.0086 (11)
C6	0.0666 (14)	0.0855 (15)	0.0373 (10)	0.0252 (13)	0.0002 (9)	0.0033 (11)
C18	0.0702 (16)	0.0680 (15)	0.0803 (18)	0.0080 (13)	−0.0010 (13)	0.0064 (13)
C12	0.0838 (18)	0.0548 (13)	0.0771 (16)	−0.0185 (13)	0.0036 (14)	−0.0131 (12)
C17	0.0653 (15)	0.0912 (18)	0.0604 (15)	0.0078 (14)	−0.0004 (12)	−0.0065 (13)
C15	0.0838 (19)	0.0674 (15)	0.0688 (16)	−0.0057 (14)	0.0116 (14)	0.0111 (12)
C20	0.0752 (19)	0.132 (3)	0.110 (2)	0.024 (2)	0.0081 (19)	−0.007 (2)
C9	0.0863 (19)	0.0603 (14)	0.096 (2)	−0.0028 (14)	−0.0098 (16)	−0.0269 (14)
C16	0.0695 (18)	0.110 (2)	0.0755 (18)	−0.0133 (17)	0.0182 (14)	0.0057 (17)
C5	0.063 (2)	0.257 (6)	0.0537 (16)	0.050 (3)	−0.0093 (14)	−0.016 (3)
C4	0.079 (2)	0.334 (8)	0.0541 (17)	−0.088 (4)	0.0068 (16)	−0.014 (3)
C3	0.168 (4)	0.169 (4)	0.0517 (16)	−0.107 (3)	0.013 (2)	−0.004 (2)

### Geometric parameters (Å, °)

**Table:**

S2—O4	1.4212 (19)	C11—H11	0.9300
S2—O3	1.4376 (16)	C8—C9	1.369 (4)
S2—N2	1.597 (2)	C8—H8	0.9300
S2—C14	1.769 (2)	C2—C3	1.511 (6)
S1—O2	1.4212 (17)	C2—H2A	0.9700
S1—O1	1.4401 (16)	C2—H2B	0.9700
S1—N1	1.6166 (19)	C6—C5	1.524 (5)
S1—C7	1.751 (3)	C6—H6	0.9800
C14—C15	1.372 (3)	C18—C17	1.371 (4)
C14—C19	1.372 (3)	C18—H18	0.9300
C7—C12	1.377 (3)	C12—H12	0.9300
C7—C8	1.380 (3)	C17—C16	1.371 (4)
N2—C6	1.475 (3)	C17—C20	1.523 (4)
N2—H102	0.9334	C15—C16	1.389 (4)
N1—C1	1.479 (2)	C15—H15	0.9300
N1—H101	0.9589	C20—H20A	0.9600
C1—C2	1.508 (3)	C20—H20B	0.9600
C1—C6	1.530 (3)	C20—H20C	0.9600
C1—H1	0.9800	C9—H9	0.9300
C10—C9	1.373 (4)	C16—H16	0.9300
C10—C11	1.384 (4)	C5—C4	1.523 (7)
C10—C13	1.497 (4)	C5—H5A	0.9700
C13—H13A	0.9600	C5—H5B	0.9700
C13—H13B	0.9600	C4—C3	1.478 (7)
C13—H13C	0.9600	C4—H4A	0.9700
C19—C18	1.379 (3)	C4—H4B	0.9700
C19—H19	0.9300	C3—H3A	0.9700
C11—C12	1.376 (4)	C3—H3B	0.9700
			
O4—S2—O3	119.38 (11)	C1—C2—H2B	109.1
O4—S2—N2	108.47 (12)	C3—C2—H2B	109.1
O3—S2—N2	105.48 (11)	H2A—C2—H2B	107.9
O4—S2—C14	107.31 (12)	N2—C6—C5	108.6 (2)
O3—S2—C14	106.81 (10)	N2—C6—C1	111.93 (17)
N2—S2—C14	109.09 (10)	C5—C6—C1	109.9 (3)
O2—S1—O1	119.01 (10)	N2—C6—H6	108.8
O2—S1—N1	108.82 (11)	C5—C6—H6	108.8
O1—S1—N1	104.81 (10)	C1—C6—H6	108.8
O2—S1—C7	107.92 (12)	C17—C18—C19	121.2 (3)
O1—S1—C7	107.91 (11)	C17—C18—H18	119.4
N1—S1—C7	107.92 (10)	C19—C18—H18	119.4
C15—C14—C19	120.6 (2)	C11—C12—C7	120.2 (2)
C15—C14—S2	120.10 (18)	C11—C12—H12	119.9
C19—C14—S2	119.28 (18)	C7—C12—H12	119.9
C12—C7—C8	119.1 (2)	C16—C17—C18	118.2 (3)
C12—C7—S1	121.16 (18)	C16—C17—C20	121.3 (3)
C8—C7—S1	119.76 (19)	C18—C17—C20	120.6 (3)
C6—N2—S2	124.00 (18)	C14—C15—C16	118.5 (2)
C6—N2—H102	117.6	C14—C15—H15	120.7
S2—N2—H102	112.8	C16—C15—H15	120.7
C1—N1—S1	119.21 (15)	C17—C20—H20A	109.5
C1—N1—H101	118.5	C17—C20—H20B	109.5
S1—N1—H101	109.1	H20A—C20—H20B	109.5
N1—C1—C2	109.21 (18)	C17—C20—H20C	109.5
N1—C1—C6	108.92 (17)	H20A—C20—H20C	109.5
C2—C1—C6	112.2 (2)	H20B—C20—H20C	109.5
N1—C1—H1	108.8	C8—C9—C10	122.6 (2)
C2—C1—H1	108.8	C8—C9—H9	118.7
C6—C1—H1	108.8	C10—C9—H9	118.7
C9—C10—C11	117.0 (3)	C17—C16—C15	121.9 (3)
C9—C10—C13	121.8 (2)	C17—C16—H16	119.1
C11—C10—C13	121.2 (3)	C15—C16—H16	119.1
C10—C13—H13A	109.5	C4—C5—C6	112.6 (3)
C10—C13—H13B	109.5	C4—C5—H5A	109.1
H13A—C13—H13B	109.5	C6—C5—H5A	109.1
C10—C13—H13C	109.5	C4—C5—H5B	109.1
H13A—C13—H13C	109.5	C6—C5—H5B	109.1
H13B—C13—H13C	109.5	H5A—C5—H5B	107.8
C14—C19—C18	119.6 (2)	C3—C4—C5	111.8 (3)
C14—C19—H19	120.2	C3—C4—H4A	109.3
C18—C19—H19	120.2	C5—C4—H4A	109.3
C12—C11—C10	121.5 (2)	C3—C4—H4B	109.3
C12—C11—H11	119.2	C5—C4—H4B	109.3
C10—C11—H11	119.2	H4A—C4—H4B	107.9
C9—C8—C7	119.6 (2)	C4—C3—C2	111.6 (3)
C9—C8—H8	120.2	C4—C3—H3A	109.3
C7—C8—H8	120.2	C2—C3—H3A	109.3
C1—C2—C3	112.3 (3)	C4—C3—H3B	109.3
C1—C2—H2A	109.1	C2—C3—H3B	109.3
C3—C2—H2A	109.1	H3A—C3—H3B	108.0
			
O4—S2—C14—C15	−4.0 (2)	C6—C1—C2—C3	−54.0 (3)
O3—S2—C14—C15	125.1 (2)	S2—N2—C6—C5	155.9 (3)
N2—S2—C14—C15	−121.3 (2)	S2—N2—C6—C1	−82.5 (3)
O4—S2—C14—C19	178.14 (19)	N1—C1—C6—N2	170.8 (2)
O3—S2—C14—C19	−52.7 (2)	C2—C1—C6—N2	−68.2 (3)
N2—S2—C14—C19	60.8 (2)	N1—C1—C6—C5	−68.4 (3)
O2—S1—C7—C12	8.9 (2)	C2—C1—C6—C5	52.6 (3)
O1—S1—C7—C12	−120.9 (2)	C14—C19—C18—C17	−0.9 (4)
N1—S1—C7—C12	126.37 (19)	C10—C11—C12—C7	0.1 (4)
O2—S1—C7—C8	−172.6 (2)	C8—C7—C12—C11	−1.1 (4)
O1—S1—C7—C8	57.6 (2)	S1—C7—C12—C11	177.3 (2)
N1—S1—C7—C8	−55.2 (2)	C19—C18—C17—C16	0.8 (4)
O4—S2—N2—C6	−37.9 (2)	C19—C18—C17—C20	179.9 (3)
O3—S2—N2—C6	−166.88 (18)	C19—C14—C15—C16	−0.5 (4)
C14—S2—N2—C6	78.7 (2)	S2—C14—C15—C16	−178.3 (2)
O2—S1—N1—C1	51.2 (2)	C7—C8—C9—C10	0.6 (5)
O1—S1—N1—C1	179.49 (16)	C11—C10—C9—C8	−1.6 (4)
C7—S1—N1—C1	−65.69 (18)	C13—C10—C9—C8	178.0 (3)
S1—N1—C1—C2	137.8 (2)	C18—C17—C16—C15	−0.5 (4)
S1—N1—C1—C6	−99.4 (2)	C20—C17—C16—C15	−179.7 (3)
C15—C14—C19—C18	0.7 (4)	C14—C15—C16—C17	0.4 (4)
S2—C14—C19—C18	178.6 (2)	N2—C6—C5—C4	69.8 (3)
C9—C10—C11—C12	1.2 (4)	C1—C6—C5—C4	−53.0 (3)
C13—C10—C11—C12	−178.4 (3)	C6—C5—C4—C3	55.0 (4)
C12—C7—C8—C9	0.8 (4)	C5—C4—C3—C2	−54.5 (4)
S1—C7—C8—C9	−177.7 (2)	C1—C2—C3—C4	54.7 (4)
N1—C1—C2—C3	66.9 (3)		

### Hydrogen-bond geometry (Å, °)

**Table:**

*D*—H···*A*	*D*—H	H···*A*	*D*···*A*	*D*—H···*A*
N1—H101···O3^i^	0.96	2.02	2.971 (3)	171
N2—H102···O1^ii^	0.93	2.07	2.982 (3)	167
C11—H11···O4^iii^	0.93	2.55	3.214 (3)	129
C9—H9···O1^iv^	0.93	2.54	3.452 (3)	169

Symmetry codes: (i) −*x*+3/2, −*y*+1, *z*+1/2; (ii) −*x*+3/2, −*y*+1, *z*−1/2; (iii) *x*−1/2, −*y*+1/2, −*z*+1; (iv) −*x*+1, *y*+1/2, −*z*+3/2.

## References

[bb1] Ackermann, L., Bergman, R. G. & Loy, R. N. (2003). *J. Am. Chem. Soc.* **125**, 11956–15963. 10.1021/ja0361547 PMC1575919 14505417

[bb2] Bisai, A., Prasad, B. A. B. & Singh, V. K. (2005). *Tetrahedron Lett.* **46**, 7935–7939.

[bb3] Bruker (1998). *SMART* and *SAINT* Bruker AXS Inc., Madison, Wisconsin, USA.

[bb4] Costa, A. M., Garcia, C., Carroll, P. J. & Walsh, P. J. (2005). *Tetrahedron*, **61**, 6442–6446.

[bb5] Farrugia, L. J. (1997). *J. Appl. Cryst.* **30**, 565.

[bb6] Farrugia, L. J. (1999). *J. Appl. Cryst.* **32**, 837–838.

[bb7] Flack, H. D. (1983). *Acta Cryst.* A**39**, 876–881.

[bb8] Guo, C., Qiu, J. & Zhang, X. (1997). *Tetrahedron*, **53**, 4145–4158.

[bb9] Nieger, M., Josten, W. & Vogtle, F. (2004). Private communication (CCDC deposition No. 235640). CCDC, Union Road, Cambridge, England.

[bb10] Pritchett, S., Gantzel, P. & Walsh, P. J. (1999). *Organometallics*, **18**, 823–831.

[bb11] Schwarz, A. D., Chu, Z. & Mountford, P. (2010). *Organometallics*, **29**, 1246–1260.

[bb12] Sheldrick, G. M. (1996). *SADABS* University of Göttingen, Germany.

[bb13] Sheldrick, G. M. (2008). *Acta Cryst.* A**64**, 112–122. 10.1107/S0108767307043930 18156677

[bb14] Tasker, P., Squires, C., Parsons, S. & Messenger, D. (2005). Private communication (CCDC deposition No. 276825). CCDC, Union Road, Cambridge, England.

